# The association between nationality, gender, age and running performance in endurance runners: an empirical analysis of worldwide multi-distance race data from 1999 to 2024

**DOI:** 10.1038/s41598-026-46032-9

**Published:** 2026-05-23

**Authors:** Guojun Liao, Junmian Wang

**Affiliations:** School of Physical Education, Yan’an University, Yan’an, 716000 Shaanxi Province China

**Keywords:** Middle-distance running, Marathon, Eastern Africa, Japan, Kenya, Health care, Medical research, Physiology

## Abstract

In recent years, the increasing popularity of half-marathon and marathon events has promoted the significant growth of global endurance running participants. In endurance running, the gender, age and nationality of athletes are closely related to their running performance. To this end, this study, based on the official data from World Athletics (formerly IAAF) and the Association of Road Racing Statisticians (ARRS), selects three demographic variables: nationality, gender, and age, aiming to explore the performance characteristics and their correlations of global runners in four different distance endurance running events: 5 km, 10 km, half marathon, and marathon from 1999 to 2024. The study included a total of 152,943 runners from 203 countries and regions (covering 180 countries, 21 regions and 2 special representative teams) (Male: n = 91,182; Female: n = 61,761). In terms of data analysis, this study used a mixed-effects model to analyze the trend of running performance and age over years, and used a three-way analysis of variance (nationality × gender × year) to test the interaction between variables. In addition, 8 multiple linear regression models were determined through the Akaike Information Criterion (AIC), and independent samples t-tests and Pearson correlation coefficients were used to analyze gender differences and the association between various demographic variables and performance. This study draws the following four main conclusions: (1) In all four different distance endurance running events, there were more male runners than females; (2)In the 5km, most runners were from the United States and Japan, while in the 10km, half marathon and marathon events, runners from Japan and Kenya accounted for the main share; (3) In all endurance running events except the 5km event, females were significantly older than males; (4) Males were faster than females in all distance endurance running events.

## Introduction

For planning an athlete's career, understanding the optimal age for their best sports performance is something that athletes and coaches must pay attention to. In endurance events like the marathon, peak performance shows a trend of increasing with the duration of the event or age^1^.As the performance of world records shows,peak marathon times for both men and women typically occur between the ages of 25 and 35. Studies indicate that finishing times in endurance running events increase in a non-linear (curvilinear) pattern with advancing age^2^.Age-related decline in marathon performance typically commences around age 35. Between 35 and 55, overall performance exhibits a gradual but steady decline; beyond 55, the average finishing time begins to increase markedly; and after 70, an accelerated decline in performance is observed^3^. A study of the Oslo races (2008–2018) revealed that peak performance age increases with race distance. Specifically, both genders achieved their best results at ages 18–23 in the 10 km event. In the half-marathon, this peak remained at 18–23 for women but shifted to 24–34 for men, while in the full marathon, the peak age for both sexes was further delayed to 35–39 years^4^.

In endurance running, athletes' aerobic endurance and maximal oxygen uptake are core physiological indicators for measuring performance, and are closely related to gender and peak age. Research on half marathons shows that the gender ratio of participants fluctuates with age, and women generally reach their best running performance at an earlier age than men^5^. In marathon running, the effect of aging on performance decline is significantly higher than gender differences, and this pattern also applies to recreational runners^6^. In addition, professional level has a significant impact on peak age, with elite athletes typically having a lower peak age than recreational enthusiasts. Different race distances have significant differences in the requirements for peak age. Research shows that as the race distance increases, the peak performance age tends to shift later: in 5 km and 10 km events, the peak age for top male and female athletes is concentrated in the 15–19 years old; in half marathons, this peak is delayed to the 20–24 years old group; and in full marathons, the age of the fastest runners further moves to the 25–34 years old range^7^.

The geographical origin of athletes is a key variable that cannot be ignored when assessing peak age. In long-distance endurance running (especially half marathon and marathon), the dominance of African runners has become increasingly significant^8^. Since the beginning of the twenty-first century, the landscape of world long-distance events has undergone major evolution, with the proportion of African athletes increasing significantly, especially Kenyan and Ethiopian runners have shown strong competitiveness in events from 5 km to marathon^9–11^. It is currently believed that this dominance is the result of the combined effect of multiple factors such as genetic factors (such as high levels of genetic diversity and specific genetic variations^12^), physiological characteristics (such as maximal oxygen uptake VO2max, superior running economy and muscle fiber type, etc.^11^), high-altitude training environment, cultural traditions and dietary structure^12–15^.

Existing evidence indicates that there is a correlation between geographical origin and peak age. African elite marathon runners reach their peak age significantly earlier than non-African runners, and their careers show the characteristic of "early peak, early retirement"^16^. In addition, African runners have a higher frequency of competition, and their performance at all stages of their career (first race, peak race and last race) are better than non-African runners^17^. Although different studies have minor fluctuations in the conclusion of peak age due to sample selection differences, the overall trend reflects the interactive influence of distance and geographical background on athletic peak^18^. At present, there is a lack of specialized investigation in academia on the peak age of 5 km runners and the interrelationship of their demographic characteristics. Therefore, this study includes the 5 km event in the analysis, aiming to compare the similarities and differences between middle-distance (5 km and 10 km) and long-distance (half marathon and marathon) endurance running. This study analyzed the age, gender, nationality and performance of top global athletes from 1999 to 2024, all data come from World Athletics and the Association of Road Racing Statisticians (ARRS). Therefore, based on the available data, we first make the following hypothesis: in half marathon and marathon races, the fastest peak performance runners are from East Africa, but not in 5km and 10km races^19^. Based on this, and combined with the influence pattern of age on peak performance in endurance running, we further propose the following hypothesis: in 5km and 10km events, the average age of East African runners is also significantly lower than that of runners from other regions.

## Methods

### Study design

To test the above proposed hypotheses, we according to the publicly available data on the official websites of World Athletics (formerly IAAF) and the Association of Road Racing Statisticians (ARRS), statistically analyzed all records of 5km, 10km, half marathon and marathon events listed during the period from 1999 to 2024, and then based on the obtained data, statistically analyzed the relationship between athletes' nationality, gender, age and running performance.

### Data source

The data of this study come from the official public data of World Athletics (formerly IAAF) and the Association of Road Racing Statisticians (ARRS). We systematically retrieved all finisher data for 5km, 10km, half marathon and marathon events from the IAAF website during the period from 1999 to 2024, and used ARRS data to supplement it, and then analyzed the relationship between athlete nationality, gender, age and running performance. This study has been approved by the Research Ethics Committee of Yan'an University (Shaanxi Province). Since all data are public information, the requirement for participants' informed consent is exempted.

### Data selection and processing

Regarding sample selection, data for the 10 km, half-marathon, and marathon events were largely complete from the World Athletics database, with minimal missing values. However, for the 5 km event, only data from 2018 to 2024 were available from World Athletics, with no records from 1999 to 2017. To address this gap, supplementary data for the 5 km event were collected from the Association of Road Racing Statisticians (ARRS) website. Due to inconsistencies in data formats across events, manual extraction was employed to ensure accuracy. During data cleaning, invalid codes and missing values in the nationality field were addressed; the sex field was generally error-free; age was recorded with two decimal places for computational consistency; and records with DQ (disqualified), DNF (did not finish), DNS (did not start), or missing values in the performance field were excluded from statistical analysis.The final dataset comprised 152,943 runners (91,182 men; 61,761 women), distributed as follows: 15,901 in the 5 km (8,715 men, 7,186 women), 38,545 in the 10 km (23,789 men, 14,756 women), 44,825 in the half-marathon (27,789 men, 17,036 women), and 53,672 in the marathon (30,889 men, 22,783 women). Prior to analysis, performance cut-off times were established for each event (uniform for both sexes): 25:00 (MM:SS) for the 5 km, 50:00 (MM:SS) for the 10 km, 1:40:00 (HH:MM:SS) for the half-marathon, and 3:20:00 (HH:MM:SS) for the marathon. Subsequently, data from two neutral teams (the Refugee Team and the Neutral Athletes Team) across all events were excluded to prevent potential confounding effects.

### Statistical analysis

All statistical analyses were performed using IBM SPSS Statistics v.22.0 (SPSS, Chicago, IL). Descriptive statistical information can be found in the appendix table, including the participation proportion of athletes from different nationalities, gender distribution percentage, and the mean and standard deviation of age and running performance. To explore the effects of gender and nationality on running performance and age between 1999 and 2024, we established a mixed-effects model, in which individual athletes were set as random effects, and nationality, gender and competition year were set as fixed effects, aiming to analyze the changing trends of running performance and age over years and test the interaction between fixed effects. According to different genders and four running distances, the Akaike Information Criterion (AIC) was used to select the optimal model, and finally 8 multiple linear regression models were determined for data analysis. The age of athletes was corrected to avoid misjudging the age effect as a time effect. For these 8 models, a three-way ANOVA (nationality × gender × year) was used to test the interaction effects of these variables on athletic performance. The correlation between nationality and gender was analyzed by one-way ANOVA (reporting F value and P value) and chi-square test (χ^2^), that is, to test the difference in gender ratio among athletes of different nationalities. Finally, partial eta squared (η^2^) was calculated as the effect size, and classified into four categories according to its size: very small (η^2^ < 0.01), small (0.01 ≤ η^2^ < 0.06), medium (0.06 ≤ η^2^ < 0.14), large (η^2^ ≥ 0.14).Subsequently, independent samples t-tests were used to examine sex-based differences in performance and age. Effect sizes were evaluated with Cohen’s d and interpreted as: trivial (d ≤ 0.2), small (0.2 < d ≤ 0.6), moderate (0.6 < d ≤ 1.2), large (1.2 < d ≤ 2.0), and very large (d > 2.0)^20,21^.It should be emphasized that the "main effect" used in the methods and results sections of this paper is composed of strict statistical terminology and does not imply any study of causal relationships between variables. In addition, Pearson correlation coefficient (r) was used to analyze the relationship between nationality, gender, age and running performance of runners in four different distances. The interpretation of the correlation coefficient refers to Cohen's conventional criteria: negligible (r < 0.20), weak (0.20 ≤ r < 0.40), moderate (0.40 ≤ r < 0.60), strong (0.60 ≤ r < 0.80), very strong (0.80 ≤ r < 1.00). The statistical significance level was set at α = 0.05, and all data were expressed as mean ± standard deviation.It should be emphasized that the "main effect" used in the methods and results sections of this paper is composed of strict statistical terminology and does not imply any study of causal relationships between variables. In addition, Pearson correlation coefficient (r) was used to analyze the relationship between nationality, gender, age and running performance of runners in four different distances. The interpretation of the correlation coefficient refers to Cohen's conventional criteria: negligible (r < 0.20), weak (0.20 ≤ r < 0.40), moderate (0.40 ≤ r < 0.60), strong (0.60 ≤ r < 0.80), very strong (0.80 ≤ r < 1.00). The statistical significance level was set at α = 0.05, and all data were expressed as mean ± standard deviation^20,21^.

## Results

### Running performance is affected by gender and race distance

Data analysis showed that gender and race distance had significant effects on athletes' running speed. Among the four race distances, the 5 km event had the fastest overall speed and was the only one with an average speed (male + female) exceeding 20 km/h.

In all events, male athletes were significantly faster than females, with an average difference of more than 2 km/h. Specifically: 5 km (Men 14:14 ± 00:37 vs. Women 15:51 ± 00:56(minutes:seconds), P < 0.001, d = -2.09), 10 km (Men 30:49 ± 02:51 vs. Women 35:11 ± 02:16(minutes:seconds), P < 0.001, d = -1.66), half marathon (Men 1:06:15 ± 0:03:46 vs. Women 1:17:15 ± 0:05:16(hours:minutes:seconds), P < 0.001, d = -2.51) and marathon (Men 2:22:36 ± 0:09:20 vs. Women 2:46:26 ± 0:12:44(hours:minutes:seconds), P < 0.001, d = -2.18). Complete data see appendix (Table 1).

**Table 1:** The coefficients(B) and standard errors(SE) of the multiple regression model for age, classified by nationality and gender.

	Β	SE	β	T	P
*5 km*					
Nationality	0.030	0.005	0.140	5.728	< 0.001
Sex	-0.029	0.262	-0.002	-0.111	0.912
Sex × nationality	-0.010	0.003	-0.091	-3.113	0.002
*10km*					
Nationality	-0.014	0.003	-0.079	-5.131	< 0.001
Sex	-0.203	0.158	-0.014	-1.288	0.198
Sex × nationality	0.009	0.002	0.093	5.085	< 0.001
*21.1km*					
Nationality	-0.010	0.002	-0.063	-4.405	< 0.001
Sex	0.780	0.155	0.054	5.023	< 0.001
sex × nationality	0.009	0.002	0.103	5.849	< 0.001
42.2km					
Nationality	0.007	0.002	0.041	3.058	0.002
Sex	0.858	0.132	0.062	6.498	< 0.001
sex × nationality	0.003	0.001	0.032	1.988	0.047

B, not standardized regression coefficient; SE, standard error of B; β, standardized regression coefficient; T, result (t-value) of t-test of regression coefficient; P, P-value of t-test of regression coefficient.

### Nationality, sex, age, and running speed differences in the 5 km event

In the 5 km event, the top five countries in terms of number of participants were the United States, Japan, Kenya, France and Australia, and their participants accounted for 62.11% of the total number. Among them, the United States had the highest proportion of participants, accounting for 28.5% of the total number (male 14.41%, female 14.11%); according to the gender composition of the total number of American participants, males accounted for 50.52% and females accounted for 49.48% Table 2.

**Table 2:** The coefficients(B) and standard errors(SE) of the multiple regression model for running performance classified by nationality, gender and age.

	B	SE	β	T	P
*5 km*					
Nationality	-0.177	0.031	-0.097	-5.719	< 0.001
Sex	84.563	1.562	0.628	54.150	< 0.001
Interaction sex × nationality	0.179	0.020	0.180	8.930	< 0.001
*10km*					
Nationality	0.295	0.059	0.060	4.969	< 0.001
Sex	273.060	3.476	0.653	78.562	< 0.001
Interaction sex × nationality	-0.145	0.040	-0.052	-3.631	< 0.001
*21.1km*					
Nationality	-0.688	0.088	-0.072	-7.788	< 0.001
Sex	625.700	5.888	0.732	106.276	< 0.001
Interaction sex × nationality	0.383	0.058	0.074	6.594	< 0.001
*42.2km*					
Nationality	0.507	0.206	0.022	2.459	0.014
Sex	1364.247	12.629	0.700	108.021	< 0.001
Interaction sex × nationality	0.713	0.132	0.059	5.407	< 0.001

Age analysis revealed no significant difference in the overall mean age between men and women in the 5 km event (men: 25.94 ± 8.27 vs. women: 25.25 ± 7.14 years; P = 0.910, d = 0.09). Among male runners, the five countries with the highest participation counts remained consistent with the overall top five. Greek runners showed the youngest mean age (22.04 ± 3.56 years), whereas Czech runners had the oldest mean age (29.94 ± 8.28 years). For female runners, the nationality ranking differed slightly: the United States and Japan remained first and second, while France, Kenya, and China placed third to fifth. Chinese female runners exhibited the youngest mean age (21.72 ± 2.78 years), and Czech female runners the oldest (31.29 ± 7.82 years).

In the 5 km event, there were significant sex differences in race performance among runners of different nationalities (men: P < 0.001, η^2^ = 0.166; women: P < 0.001, η^2^ = 0.216). Among male runners, except for runners from Ethiopia, Qatar, Kenya and Uganda, Bahrain runners (13:28 ± 00:33 min:seconds) were faster than all other nationalities, while except for Greek, Danish, French and Romanian nationalities, Czech runners were slower than all other nationalities. Among female runners, except for Danish, Ethiopian, Slovenian, Romanian nationalities, British runners (14:43 ± 00:41 min:seconds) were faster than all other nationalities, while except for New Zealand, Brazilian, Peruvian and Hungarian nationalities, Swiss runners (16:58 ± 01:09 min:seconds) were slower than all other nationalities (Table 3).

**Table 3:** The coefficients(B) and standard errors(SE) of the multiple regression model for running performance classified by nationality, gender and age.

	B	SE	β	T	P
*5 km*					
Nationality	0.066	0.010	0.036	6.788	< 0.001
Sex	157.913	2.501	1.172	63.140	< 0.001
Age	4.296	0.138	0.499	31.234	< 0.001
Sex × age	-2.362	0.094	-0.589	-25.153	< 0.001
*10km*					
Nationality	0.098	0.019	0.020	5.237	< 0.001
Sex	275.644	6.440	0.659	42.802	< 0.001
Age	7.117	0.326	0.252	21.804	< 0.001
sex × age	-0.590	0.219	-0.052	-2.697	0.007
*21.1km*					
Nationality	-0.180	0.026	-0.019	-6.845	< 0.001
Sex	601.965	10.117	0.704	59.498	< 0.001
Age	13.787	0.496	0.232	27.825	< 0.001
Sex × age	1.156	0.334	0.052	3.462	0.001
*42.2km*					
Nationality	1.176	0.061	0.052	19.223	< 0.001
Sex	1178.229	25.125	0.605	46.895	< 0.001
Age	26.398	1.215	0.188	21.735	< 0.001
Sex × age	6.440	0.762	0.134	8.447	< 0.001

### Nationality, sex, age, and running speed differences in the 10 km event

In the 10 km event, the five countries with the largest number of participants were France, Kenya, Spain, the United States and Japan, which together accounted for 61.37% of the total participants. Among them, French participants accounted for the highest proportion (8.9%), and their gender composition was mainly male (68.28%), with females accounting for 31.72%.

In terms of age, the average age of male runners in the 10 km event was significantly smaller than that of females (27.26 ± 7.02 vs. 28.76 ± 7.44 years, P < 0.001, d = -0.22). Among the five countries with the largest number of male participants, the top three were consistent with the overall ranking, while Italy and the United States ranked fourth and fifth respectively. Among them, the average age of Somali runners was the smallest (22.45 ± 3.36 years), and the average age of Welsh runners was the largest (35.24 ± 6.80 years). The nationality ranking of female runners changed slightly, with the top five being the United States, Japan, France, Kenya and Spain. The average age of Chinese runners was the smallest (22.47 ± 3.84 years), and the average age of Welsh runners was the largest (35.92 ± 5.55 years).

In the 10km event, the running performance of men (P < 0.001, η^2^ = 0.064) and women (P < 0.001, η^2^ = 0.233) of different nationalities was different. Among male runners, except for runners from Israel, Uganda, Ethiopia and Kenya, Tanzanian runners (29:16 ± 00:59 min:seconds) surpassed all other nationalities, becoming the fastest in terms of average running performance among all nationalities. Except for Latvian, American, Croatian and Japanese nationalities, Serbian runners were slower than all other nationalities. Among female runners, except for Ethiopian, Bahraini, Kenyan and Japanese nationalities, Ugandan runners (32:49 ± 02:16 min:seconds) were faster than all other nationalities. In the ranking of the longest average running time by nationality, except for Serbian, Icelandic, Estonian and Greek nationalities, Uruguayan runners (37:50 ± 00:49 min:seconds) were slower than all other nationalities (Table 4).

**Table 4:** The coefficients(B) and standard errors(SE) of the multiple regression model for running performance classified by calendar year and gender.

	B	SE	β	T	P
*5 km*					
Calendar year	0.146	0.058	0.014	2.531	0.011
Sex	91.858	0.881	0.682	104.212	< 0.001
Sex × nationality × calendar Year	3.579	< 0.001	0.072	11.018	< 0.001
*10km*					
Calendar year	3.732	0.133	0.111	28.031	< 0.001
Sex	255.432	1.953	0.611	130.810	< 0.001
Sex × nationality × calendar year	3.916	< 0.001	0.028	6.015	< 0.001
*21.1km*					
Calendar year	3.138	0.211	0.045	14.891	< 0.001
Sex	662.008	3.124	0.774	211.877	< 0.001
Sex × nationality × calendar year	-6.507	< 0.001	-0.003	-0.690	0.490
*42.2km*					
Calendar year	5.106	0.529	0.028	9.660	< 0.001
Sex	1337.388	6.827	0.686	195.902	< 0.001
Sex × nationality × calendar year	0.001	< 0.001	0.087	24.847	< 0.001

### Nationality, sex, age, and running speed differences in the half-marathon event

In the half-marathon event, the top five countries by participant count were Japan, Kenya, the United States, Ethiopia, and Italy, collectively comprising 45.89% of all participants. Japan contributed the largest share (17.7%), with male runners forming the majority (76.71%) and female runners comprising 23.29%.

Age analysis revealed that male runners were significantly younger than female runners in the half-marathon event (28.5 ± 6.84 vs. 30.11 ± 7.14 years; P < 0.001, d = -0.33), with age distribution varying by nationality. Among male runners, the top three countries by participant count remained consistent with the overall ranking, while France and Ethiopia ranked fourth and fifth, respectively. Qatari male runners showed the youngest mean age (23.66 ± 3.52 years), whereas English male runners had the oldest mean age (37.29 ± 6.48 years). The nationality ranking differed among female runners, with Kenya, Japan, the United States, Ethiopia, and Italy comprising the top five. Ethiopian female runners exhibited the youngest mean age (24.02 ± 3.93 years), while English female runners had the oldest mean age (35.51 ± 4.89 years).

In the half marathon event, the running performance of men (P < 0.001, η^2^ = 0.275) and women (P < 0.001, η^2^ = 0.308) of different nationalities was different. Among male runners, except for runners from Ethiopia, Kenya, Eritrea and Uganda, Brunei runners (1:02:51 ± 0:02:24 h:minutes:seconds) surpassed all other nationalities, becoming the fastest in terms of average running performance among all nationalities. Except for runners from Thailand, Hong Kong, Greece and England and other countries and regions, Croatian runners were slower than all other nationalities. Among female runners, except for runners from Ethiopia, Kenya, Burundi and Tanzania and other countries and regions, Bahrain runners (1:10:48 ± 0:03:12 h:minutes:seconds) were faster than all other nationalities. In the ranking of the longest average running time by nationality, except for runners from Iceland, Greece, Uzbekistan and Serbia and other countries and regions, Thai runners (37:50 ± 00:49 h:minutes:seconds) were slower than all other nationalities Table 5.

**Table 5:** The coefficients(B) and standard errors(SE) of the multiple regression model for runner’s ages classified by calendar year and nationality.

	B	SE	β	T	P
*5 km*					
Calendar year	0.037	0.010	0.030	3.779	< 0.001
Nationality	0.015	0.002	0.072	8.985	< 0.001
Sex × calendar year	< 0.001	< 0.001	-0.048	-6.038	< 0.001
*10km*					
Calendar year	-0.028	0.006	-0.023	-4.518	< 0.001
Nationality	-0.001	0.001	-0.008	-1.477	0.140
Sex × calendar year	< 0.001	< 0.001	0.034	6.728	< 0.001
*21.1km*					
Calendar year	-0.014	0.006	-0.012	-2.477	0.013
Nationality	0.002	0.001	0.015	3.158	0.002
Sex × calendar year	0.001	< 0.001	0.111	23.609	< 0.001
*42.2km*					
Calendar year	0.107	0.006	0.083	19.312	< 0.001
Nationality	0.012	0.001	0.074	17.119	< 0.001
Sex × calendar year	0.001	< 0.001	0.082	19.077	< 0.001

### Nationality, sex, age, and running speed differences in the marathon event

In the marathon event, the five countries with the largest number of participants were Japan, Kenya, the United States, Ethiopia and China, which together accounted for 59.36% of the total participants. Among them, Japanese participants accounted for the highest proportion (17.9%), and their gender composition was 61.53% male and 38.47% female.

**Fig. 1:**
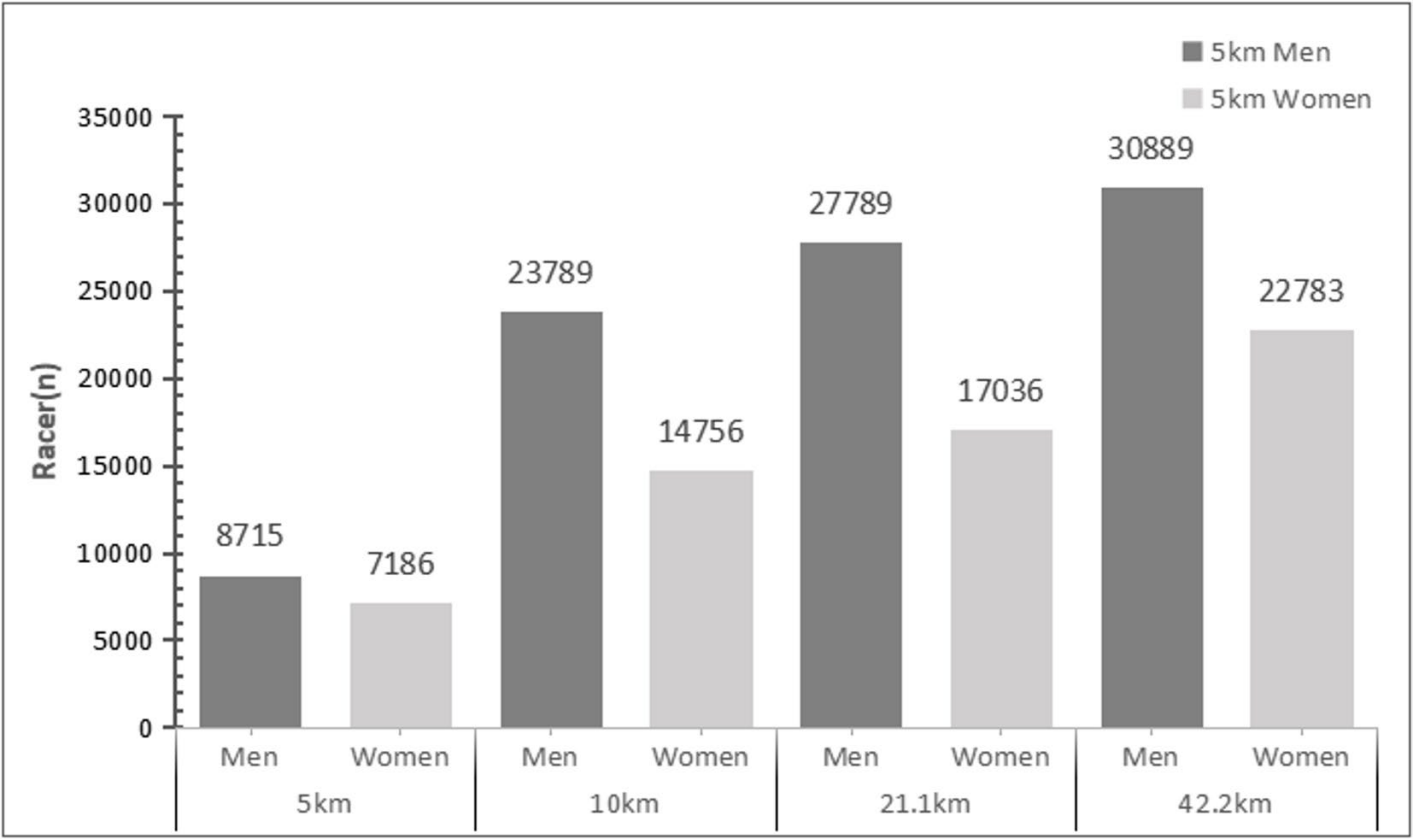
The number of runners classified by race distance and gender.

Age analysis revealed that male runners were significantly younger than female runners in the marathon event (31.67 ± 6.28 vs. 32.8 ± 7.49 years; P < 0.001, d = -0.27), with age distribution varying by nationality. Among male runners, the top five countries by participant count remained consistent with the overall ranking, with Japan contributing the highest proportion (19.11%). North Korean male runners showed the youngest mean age (25.88 ± 2.87 years), whereas Portuguese male runners had the oldest mean age (36.63 ± 7.27 years). The nationality ranking differed among female runners, with the United States, Japan, Ethiopia, Kenya, and China comprising the top five. North Korean female runners exhibited the youngest mean age (24.31 ± 3.52 years), while Italian female runners had the oldest mean age (37.90 ± 7.70 years) Fig. 1.

**Fig. 2:**
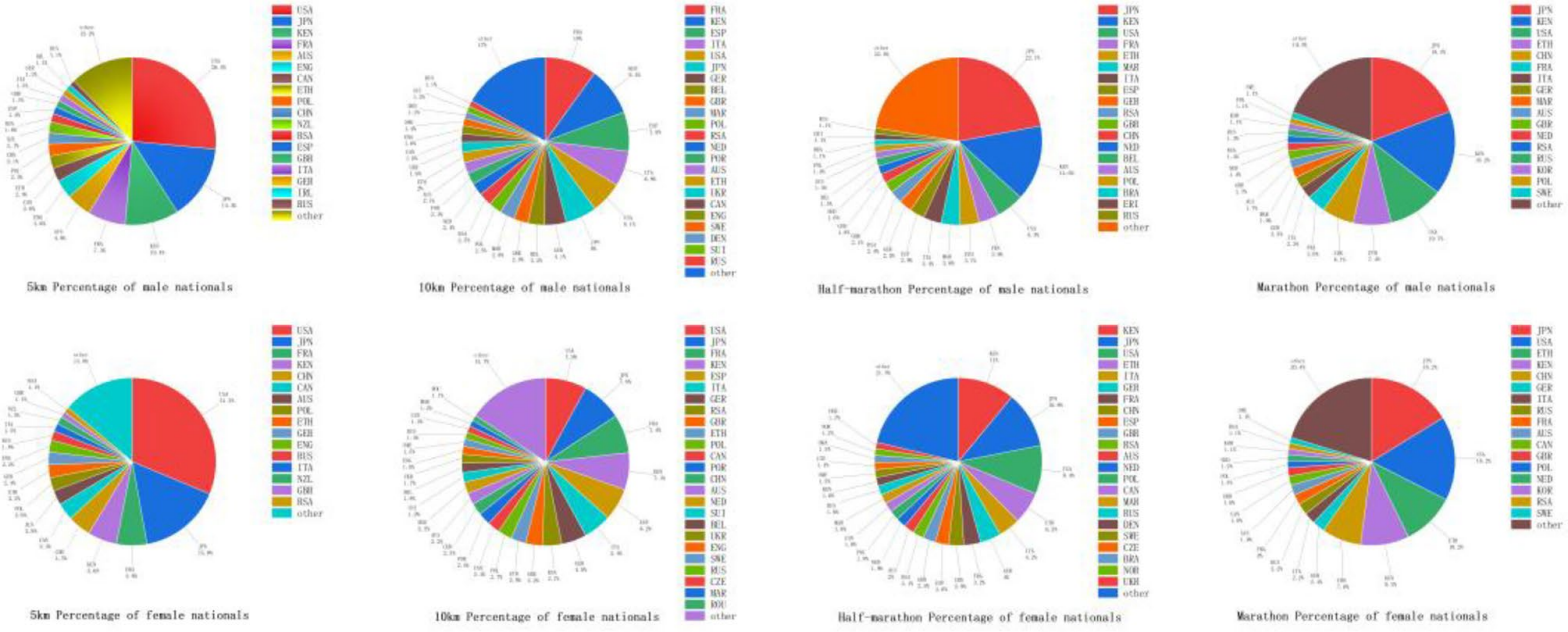
The proportion of runners' nationalities by race distance and gender. Countries with a proportion of less than 1% are included in "Others".

In the marathon event, the running performance of men (P < 0.001, η^2^ = 0.281) and women (P < 0.001, η^2^ = 0.292) of different nationalities was different. In the male runners of the marathon event, except for runners from Ethiopia, Eritrea, Uganda and Kenya, as in the 5km and half marathon events, Bahraini runners' average running performance (2:12:07 ± 0:05:13 h:minutes:seconds) surpassed all other nationalities, becoming the fastest in terms of average running performance among all nationalities. Except for participants from Slovenia, Hong Kong, Hungary and Taiwan and other countries or regions, as in the half marathon, Croatian runners were slower than all other nationalities. Among female runners, except for runners from Ethiopia, North Korea, Kenya and Namibia and other countries and regions, as in the half marathon event, Bahraini runners' running performance (2:29:24 ± 0:07:45 h:minutes:seconds) was faster than all other nationalities. In the ranking of the longest average running time by nationality, except for runners from Taiwan, Thailand, Hong Kong and India and other countries and regions, Slovenian runners' running performance (2:59:03 ± 0:09:02 h:minutes:seconds) was slower than all other nationalities Figs. 2.and Table 6.

**Table 6:** The coefficients(B) and standard errors(SE) of the multiple regression model for runner’s ages classified by calendar year and gender.

	β	SE(β)	Stand β	T	P
*5 km*					
calendar year	0.033	0.010	0.027	3.420	0.001
sex	-1.298	0.148	-0.083	-8.768	< 0.001
sex × nationality × calendar year	4.079	< 0.001	0.071	7.477	< 0.001
*10km*					
calendar year	-0.026	0.006	-0.022	-4.280	< 0.001
sex	0.488	0.090	0.033	5.446	< 0.001
sex × nationality × calendar year	9.195	< 0.001	0.002	0.308	0.758
*21.1km*					
calendar year	-0.012	0.006	-0.010	-2.127	0.033
sex	1.369	0.083	0.095	16.580	< 0.001
sex × nationality × calendar year	1.222	< 0.001	0.028	4.903	< 0.001
*42.2km*					
calendar year	0.106	0.006	0.082	19.215	< 0.001
sex	0.501	0.071	0.036	7.032	< 0.001
sex × nationality × calendar year	3.664	< 0.001	0.086	16.753	< 0.001

### Age and race time by calendar year and race distance

**Fig. 3:**
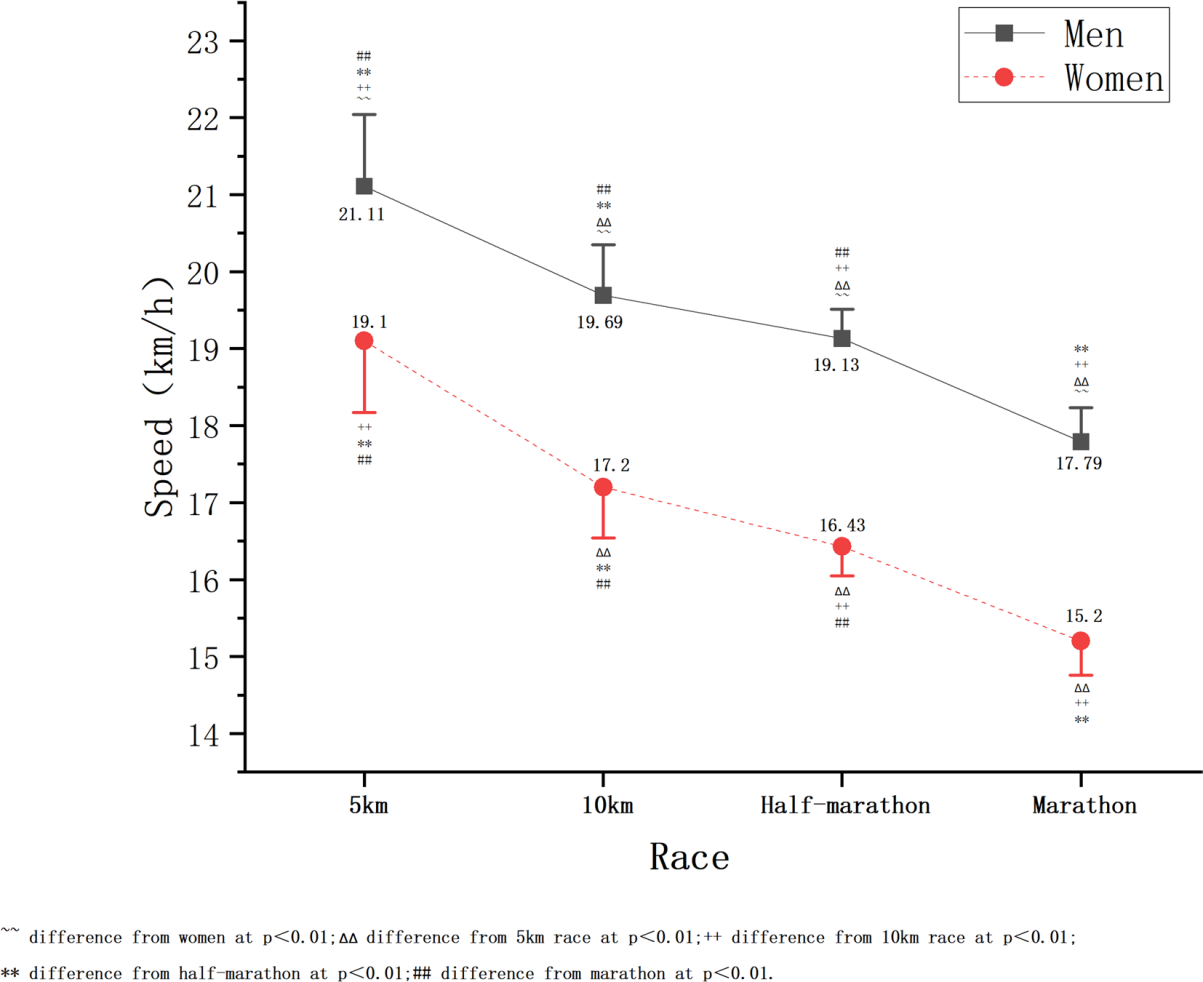
Running speeds classified by race distance and gender.

**Fig. 4:**
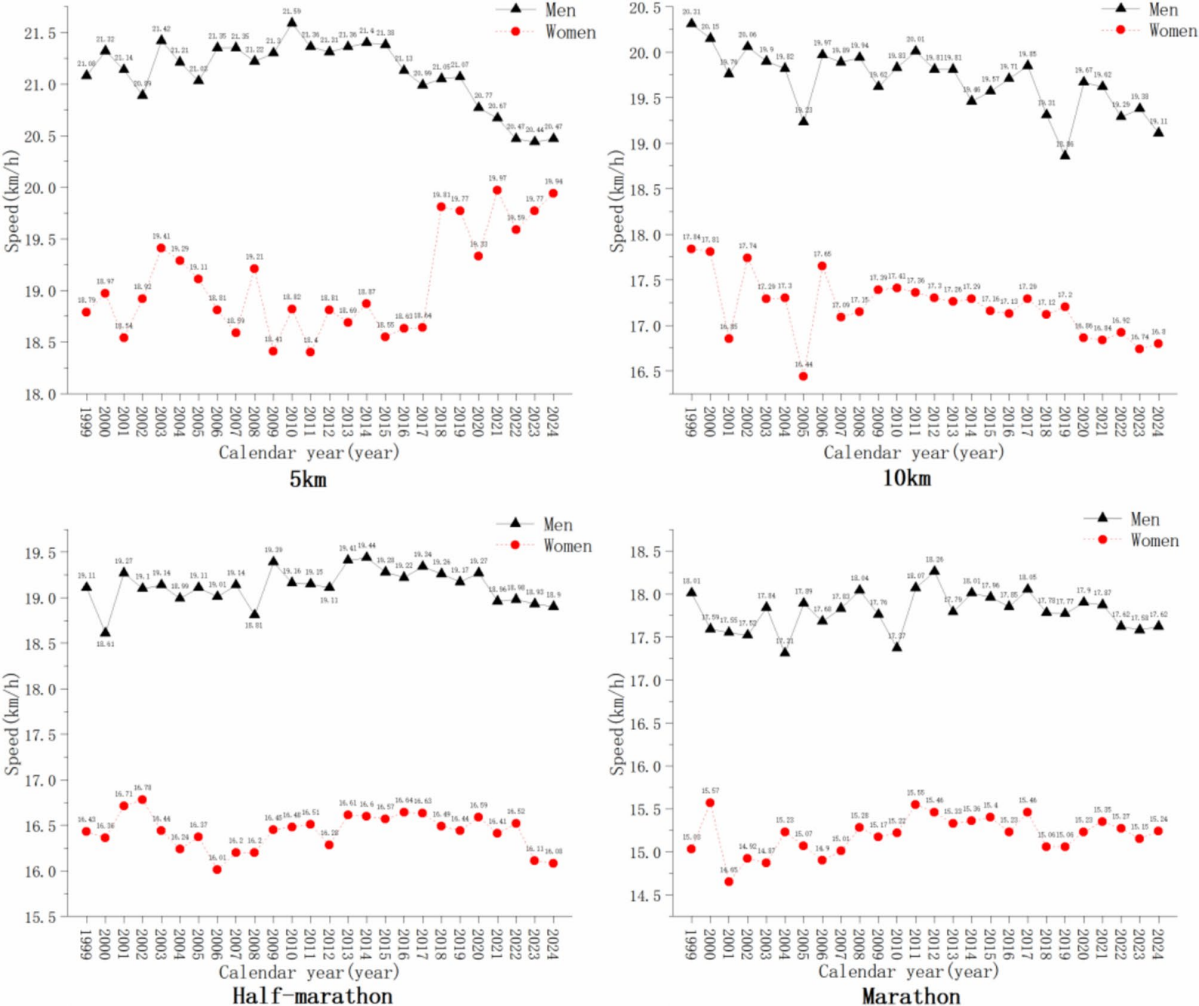
Running speed classified by calendar year and race distance.

In these four different distance endurance running events, running performance showed significant changes with calendar year (P ≤ 0.011; Table 4). For example, interaction between gender and calendar year was observed in 5km, 10km and marathon events (P ≤ 0.001), but not in the half marathon event (P > 0.05). According to (Table 12), participant age also changed significantly with calendar year (P < 0.05). The changes in participation, age and race time of the subjects in this experiment are shown in Figs. 3 and 4 by calendar year, gender and race distance, respectively. In addition, according to the three-way ANOVA results, nationality [F(202, 151173) = 12.255,η^2^ = 0.016,P < 0.001], gender [F(1, 151173) = 69.312,η^2^ < 0.01,P < 0.001], age group [F(9, 151173) = 116.270,η^2^ = 0.01,P < 0.001] all had significant main effects on endurance running of different distances. Furthermore, according to the interaction effects of the three-way ANOVA, there were significant interactions between nationality × gender [F(140, 151173) = 1.853,η^2^ < 0.01,P < 0.001] and age group × nationality [F(845, 151173) = 6.303,η^2^ = 0.034,P < 0.001], while there was no interaction between gender × age group [F(9, 151173) = 1.421,η^2^ < 0.01,P = 0.173]. Finally, the interaction between nationality × gender × age group [F(553, 151173) = 2.117,η^2^ = 0.01,P < 0.001] was statistically significant. Here, we also conducted additional post-hoc tests to evaluate the performance of men and women in these 4 different distance endurance running events (as shown in Fig. 3) Table 7.

**Table 7:** The number of participants classified by race distance, gender and age group.

	5km race					10km race					Half-marathon					Marathon			
**Age groups**	**Men**	**Women**	**Total**	**MWR**		**Men**	**Women**	**Total**	**MWR**		**Men**	**Women**	**Total**	**MWR**		**Men**	**Women**	**Total**	**MWR**
**10–20**	918	1114	2032	0.82		1396	978	2374	1.43		1465	661	2126	2.22		146	232	378	0.63
**20–25**	4121	3289	7410	1.25		7065	4211	11,276	1.68		7820	3695	11,515	2.12		3742	3009	6751	1.24
**25–30**	2367	1653	4020	1.43		7715	4315	12,030	1.79		8826	5187	14,013	1.7		9930	6087	16,017	1.63
**30–35**	855	653	1508	1.31		4594	2767	7361	1.66		5871	3779	9650	1.55		9238	5820	15,058	1.9
**35–40**	184	226	410	0.81		1815	1332	3147	1.36		2397	2172	4569	1.1		4878	3833	8711	1.27
**40–45**	51	128	179	0.4		712	706	1418	1.01		882	978	1860	0.9		2007	2162	4169	0.93
**45–50**	18	26	44	0.69		204	242	446	0.84		210	344	554	0.61		614	1111	1725	0.55
**50–55**	14	23	37	0.61		64	77	141	0.83		103	103	206	1		211	371	582	0.57
**55–60**	18	10	28	1.8		40	43	83	0.93		55	60	115	0.92		63	125	188	0.50
**60 + **	169	64	233	2.64		184	85	269	2.16		160	57	217	2.81		60	33	93	1.82
**Total**	8715	7186	15,901	1.21		23,789	14,756	38,545	1.61		27,789	17,036	44,825	1.63		30,889	22,783	53,672	1.36

MWR = Ratio of men to women

### Relationship between runner age and running performance across events

Notably, Kenyan and Ethiopian runners consistently ranked among the top five performers across all race distances. These two countries also maintained top-five positions in participant numbers for all four race distances, underscoring their substantial engagement in global endurance running. Furthermore, analysis of the age-performance relationship revealed distinct patterns across different race distances.

As shown in Table 8, there were significant correlations between age and running performance in all events (P < 0.001). According to observations, the 5km (men and women) and 10km (men and women) data sets were at a very weak correlation level, in which the absolute values of r for men and women were not much different. After comparing the other data sets, it was found that the half marathon (men and women) and marathon (men and women) were also at another weaker correlation level. However, in both the half marathon and marathon events, the correlation between age and running performance was greater in women than in men. Finally, the r value obtained in the 5km (women) event was special. This correlation was different from the r values obtained in other experimental groups. Its direction was negative, that is, within a certain age range (10–35 years), the older the age, the faster the running performance, which is completely opposite to other correlations.

**Table 8:** The relationship between age and running performance, classified by race distance and gender.

	Men		Women	
Race distance (km)	r	p	r	p
5	0.107	< 0.001	-0.170	< 0.001
10	0.179	< 0.001	0.171	< 0.001
21.1	0.278	< 0.001	0.293	< 0.001
42.2	0.313	< 0.001	0.368	< 0.001

## Discussion

**Fig. 5:**
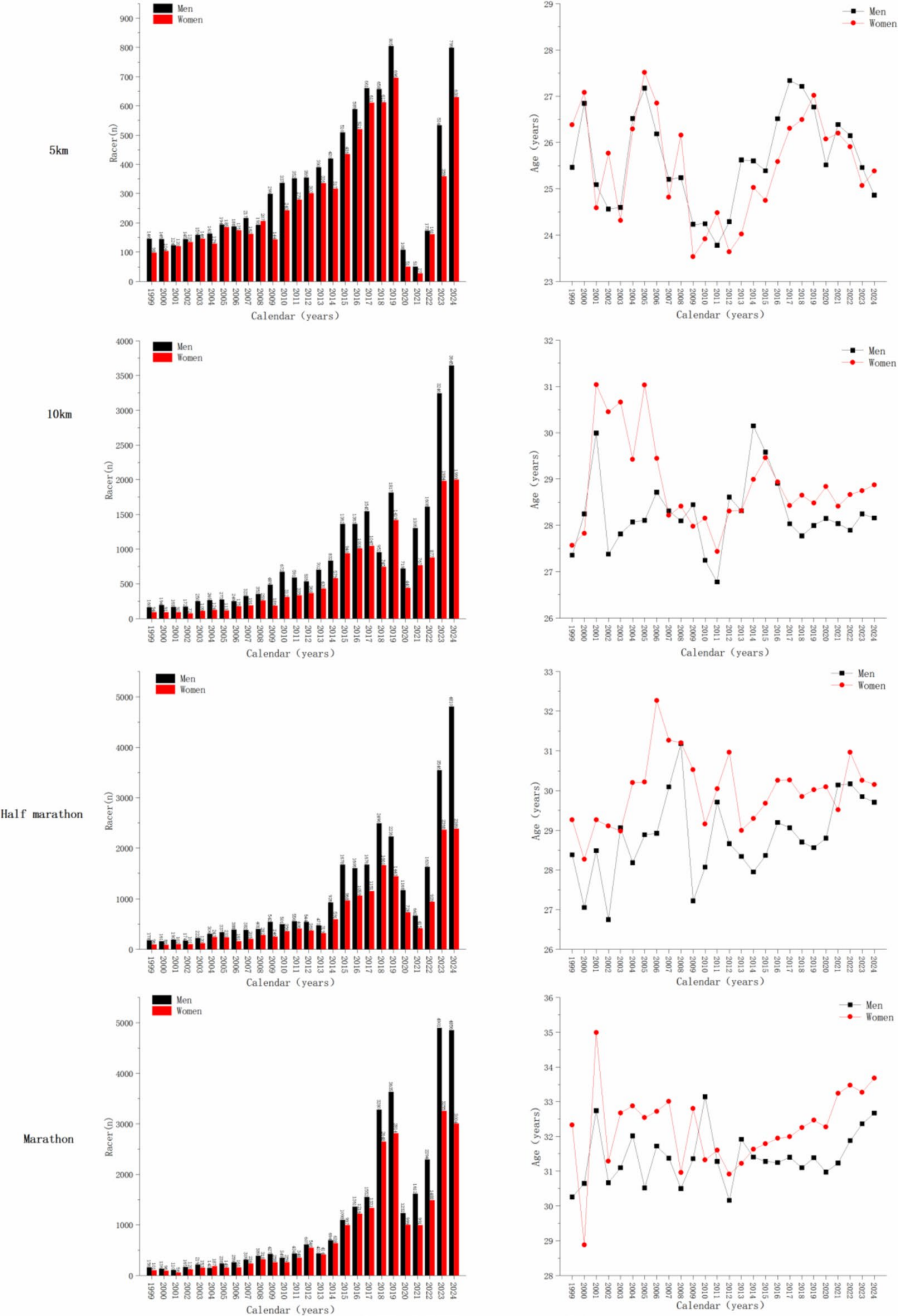
The number of participants and their ages, classified by calendar year and race distance.

This study used multiple regression models to systematically analyze the relationship between runners' nationality, gender, age and running performance. The main findings are as follows: (1) The participants in middle-distance endurance running events (5 km and 10 km) were mainly from the United States and Japan, while the participants in long-distance endurance running events (half marathon and marathon) were mainly from Japan and Kenya; (2) After comparing the nationality and region of the participating runners, it was found that except for the 5 km event, the average age of participants in other events was around 30 years old, and the average age of female runners was generally greater than that of male runners; (3) From Figs. 4 and 5, it can be clearly observed that the running speed of men was significantly faster than that of women in all events, and with the linear passage of calendar years, the speed trends of men and women in different distance events gradually became consistent after large fluctuations in the initial period. In addition, runners from East Africa performed best in long-distance events. Not only did Kenyan and Ethiopian runners perform significantly better than those from other regions, but runners from other East African countries such as Uganda and Eritrea also showed very strong competitive advantages.

First, as evidenced by the data, American and Japanese runners accounted for the highest proportions in the 5 km event, with a marked disparity in participant numbers compared to other nationalities—a pattern consistent across both sexes. Notably, Kenyan participation rates closely followed those of Japan. Considering the substantial population sizes of the US and Japan, Kenya's capacity to maintain high participation levels while achieving significantly better performance underscores its prominent status in middle- and long-distance running^22^.In the 10 km event, French and Kenyan male runners accounted for the highest proportion; while in female runners, the United States and Japan ranked in the top two. It is worth noting that the participation proportions of female runners from the US, Japan, France and Kenya were extremely close, with differences of less than one percentage point. In the half marathon men's group, Japanese and Kenyan runners far led the proportion, totaling 36.4%; in the women's group, the proportions of Kenyan and Japanese runners were basically equal (differing by only 0.1%), with the US following closely behind, and these three countries together formed a clear leading echelon. In marathon male runners, Japanese and Kenyan runners together accounted for 35.2%, with the US, Ethiopia and China forming the second echelon; in female runners, Japan and the US had the same proportion (the difference in number was only single-digit), while Ethiopia, Kenya and China formed the following second echelon, and there were significant gradient differences between each echelon.

In the distribution logic of male athletes, the leading proportion of Japanese and Kenyan athletes may reflect two different models of excellence. Kenya's advantage stems from physiological genetics and high-altitude training environment, while Japan's outstanding performance is closely related to its profound "Ekiden" culture and extremely high level of corporate team professional training system^9,23^. In the women's group, the United States and Japan show extremely high competitiveness, which may be attributed to the relatively complete women's sports support systems, university sports competition mechanisms (such as the NCAA in the United States) and the high social acceptance of women's participation in endurance sports in these countries^24^. As for the second echelon (China, Ethiopia), Ethiopian athletes are similar to Kenya, with geographical environment advantages; while the rise of Chinese athletes (entering the second echelon) reflects the explosive growth of China's marathon industry in recent years and the promotion of the "mass elite" trend to the top competitive level^9,25^.

**Fig. 6:**
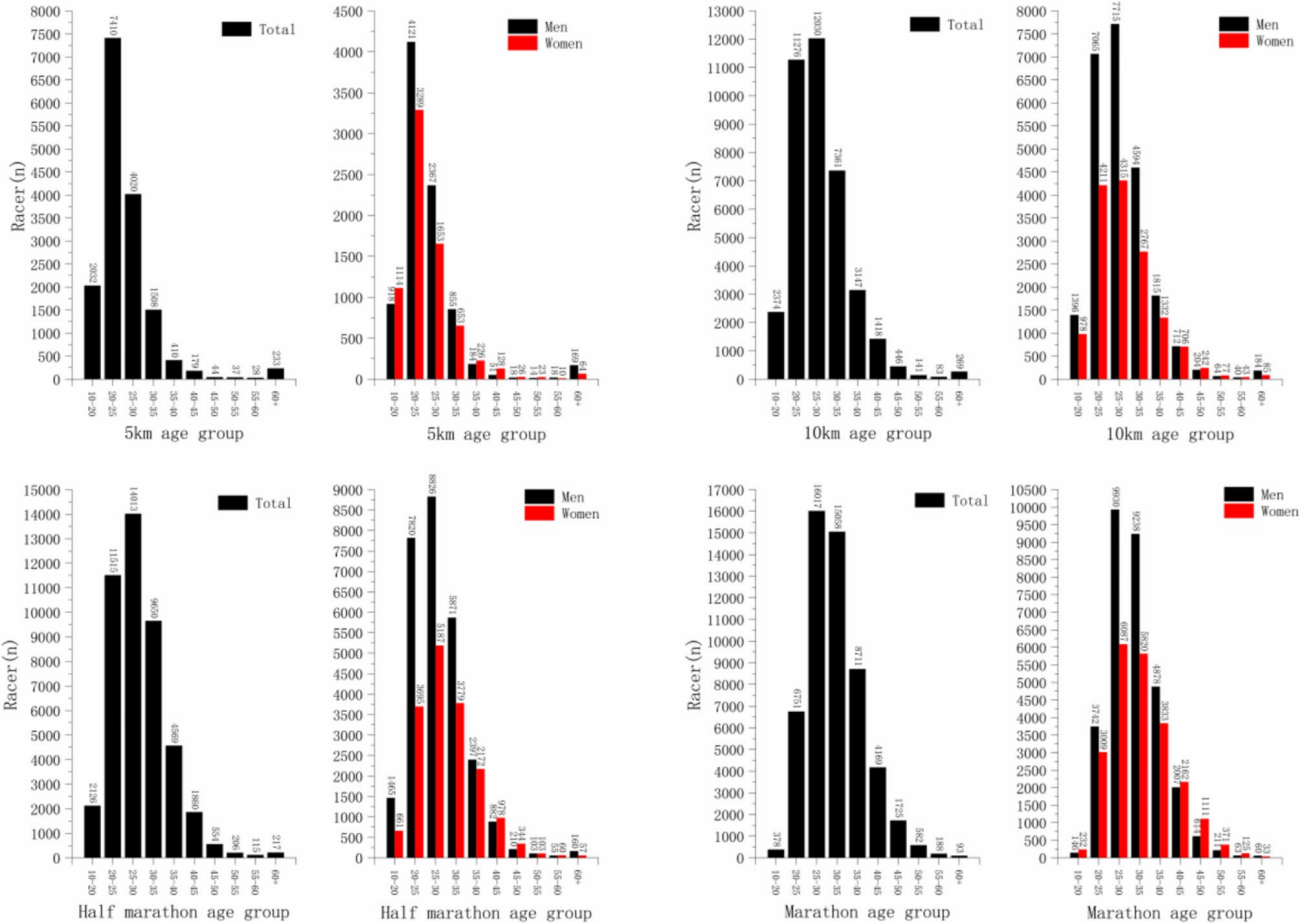
The number of participants classified by age group and race distance.

**Fig. 7:**
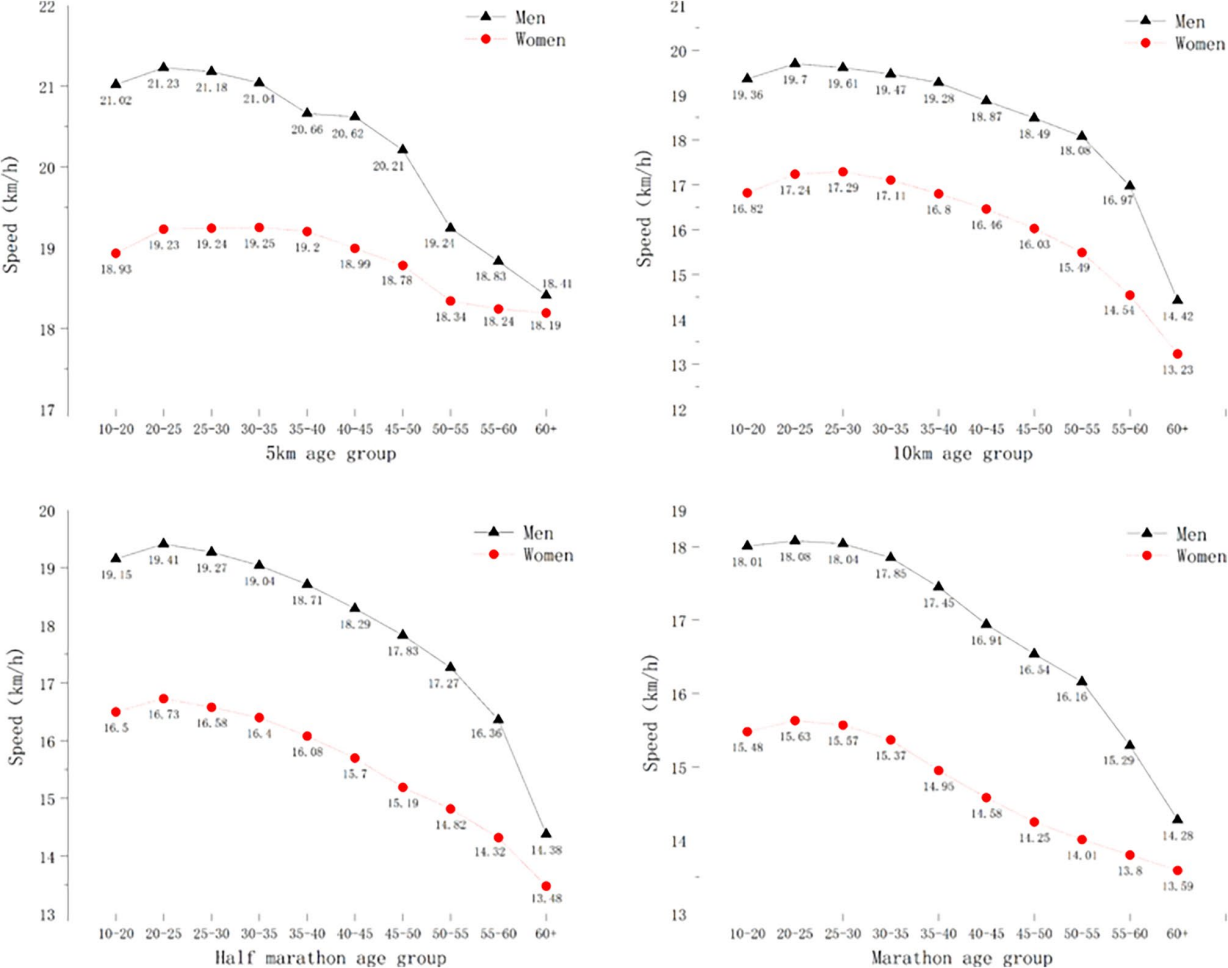
Running speed classified by age group and race distance.

Regarding the second point, analysis shows (Fig. 6) that the number of participants in middle-distance and long-distance endurance running events showed significant fluctuations and a sharp decline during 2020–2022. This trend was mainly due to the impact of the global COVID-19 pandemic, including factors such as event cancellations, public health restrictions, psychological stress and economic structure changes^26^.Age analysis shows that the age difference between male and female runners in middle-distance endurance running events is small, but as the distance increases, the difference gradually appears. In the 5 km event, the age of men and women is similar, while in the 10 km event, the age of women is gradually greater than that of men. This trend is more obvious in long-distance endurance running events: in the 26 years of marathon data studied (1999–2024), women were older than men in 23 years. In addition, male runners were faster than females in these four different distance endurance running events.In terms of the proportion of participating runners by age group (Fig. 7), 5 km event participants were mainly concentrated in the 20–25 age group (46.60%), followed by the 25–30 age group (25.28%). In the 10 km event, the 25–30 age group became the main participating age group (31.21%), followed by the 20–25 age group (29.25%). The half marathon also mainly involved the 25–30 age group (31.26%), with the 20–25 age group ranking second (25.70%). Compared with the 10 km event, the proportion of 20–25 year old participants in the half marathon decreased(The decline was 3.55 percentage points), while the proportion of 25–30 year old participants increased, but the difference was not large(Only increased by 0.05 percentage points). The main participating age group in the marathon event was 25–30 years old (29.84%), and the secondary participating age group was 30–35 years old (28.06%).It is worth noting that the main age groups of male and female participants in middle-distance endurance running events (20–25 years and 25–30 years) are consistent with the overall distribution. However, in female half marathon participants, the secondary participation age group changed from 20–25 years to 30–35 years; in the marathon event, the main age groups of both male and female participants became 25–30 years and 30–35 years. This shows that as the race distance increases, the average age of female participants grows faster than that of males, and the main age groups of all participants show an upward trend. We propose two explanations: First, the growth in participation of middle-aged and older women may be faster than that of men. Over the past few decades, the increase in female marathon participants has been mainly driven by the older age group^27^. Second, the large base of young male participants means their growth may exceed that of middle-aged and older groups. That is, the large base of male participants in the early stage of competition keeps their average age at a lower level in short-distance events, while women show a more significant age increase trend when transitioning to long-distance events^28^.

From a geographical distribution perspective, East African runners account for the highest proportion in the youngest age group (male 40%, female 45%), followed by East Asian runners (male 35%, female 40%). European runners dominate the oldest age group (75% for both men and women), among which Western Europe (male 30%, female 30%) and Southern Europe (male 20%, female 20%) account for the majority. This phenomenon may be because East Africa has a strong long-distance running culture, many young people start participating in long-distance running training from an early age, and long-distance running is an important sports and economic activity locally, attracting a large number of young runners to join^27^. The average age of marathon participants in East Asia is relatively moderate, which may be because the popularity of marathon sports in the region has gradually increased in recent years, attracting people of different age groups to participate, but overall the participation proportion of young people is relatively large^25^. Western Europe can be explained by the developed local economy, high standard of living, and greater emphasis on health and quality of life. Most people tend to participate in marathons and other sports when they reach middle age and have more time and financial resources, regarding it as a healthy lifestyle and a way of self-challenge^29^. In Southern Europe, people's lifestyle is relatively more leisurely, sports concepts and habits are different from Western Europe, the popularity of marathon sports is relatively low, and the participating population is mainly composed of middle-aged and above sports enthusiasts^30^.

Third, we examined associations between running performance and other factors. As shown in Fig. 6, speed trends for both sexes across distances demonstrated considerable initial variability before gradually converging over time. In the 5 km event, American men constituted the largest participant group, whereas Bahraini and Ethiopian runners achieved the fastest times. Notably, Bahrain's remarkable performance relies heavily on naturalized athletes—particularly middle- and long-distance runners originating from Kenya and Ethiopia—rather than domestic participation^31^.Existing surveys show that all Olympic medals historically obtained by the Kingdom of Bahrain have been won by African-born athletes. In terms of the number of participants, American women are the most numerous in each event, but British runners performed best in the 5 km event; in the 10 km event, French male participants are the most, while Tanzanian runners are the fastest; female participants are still the most in the United States, and Ugandan runners performed best. In the half marathon and marathon events, Japan and Kenya lead in the number of participants, but Bahrain and Ethiopian runners have an absolute advantage in performance, and this pattern is highly consistent in both men's and women's events.If we exclude countries that rely on naturalized athletes such as Bahrain, East African runners account for only 31.86% of the top five participation in the four distance events, but they account for as high as 85% in the top five men's results and 60% in women's, highlighting their dominance in long-distance running. It should be noted that Japanese runners have a large participation base and have significantly improved their results in recent years, but because the time span of the data in this study is long, the early data may dilute their recent performance, so it fails to fully reflect their secondary dominant position in the current long-distance running landscape^32^.

This study compiled data from runners representing 203 countries and regions (comprising 180 countries, 21 regions, and 2 neutral teams) between 1999 and 2024. Over the past two decades, performance trends across events have been shaped by differential rates of improvement and decline among male and female athletes across race distances (5 km, 10 km, half-marathon, and marathon) and age categories (e.g., 10–20, 20–25, up to 60 + years)^33^.This study shows that in the four race distances, the proportion of male participants was significantly higher than that of females; and in all distances, the possibility that female runners are older than males increases with race distance. In addition, East African runners (mainly from Kenya and Ethiopia) performed best and were the youngest in all distances, while European runners performed the slowest and had the oldest average age. The findings confirm that men perform better than women in middle- and long-distance endurance running events, but the gender performance gap gradually narrows in longer distances and higher age groups. Finally, it should be emphasized that runner performance trends are closely related to the gender ratio (MWR) as a function of age and race distance.

This study covers athletes from nearly 90% of countries and regions worldwide, with high sample representativeness, but this also constitutes the main limitation of the study: in order to maintain the completeness of national participation and performance grading, no strict distinction was made between elite and mass runners. According to international standards, changes in middle- and long-distance running speeds are the result of the combined effect of elite competitive breakthroughs and mass participation—elite athletes continuously break records through scientific training, while mass runners experience a decline in average speed due to aging, diversification of motivations, and insufficient training. In the future, with the popularization of sports science and the refinement of event stratification, this "polarization" may intensify, and the connotation of speed will become more diverse: elites pursue limits, the masses focus on health, together shaping the middle- and long-distance running sports ecology. In addition, the finding that Ethiopian male marathon runners perform better than Kenyan runners suggests that there may be differences between athletes from the two countries in terms of anthropometry, physiological characteristics, runner density, and running economy. It should be emphasized that this study focuses on athlete nationality rather than racial background. At the same time, the "borderless athlete" phenomenon (i.e., athletes changing nationality) is also a variable that needs to be considered^34,35^.Indeed, some countries resort to naturalization policies due to limited domestic talent pools in middle- and long-distance running and strong pursuit of international sporting prestige. For instance, Bahrain and Qatar—excluded from certain analyses in this study—represent typical cases of heavy reliance on naturalized athletes. Therefore, caution is warranted when generalizing findings regarding the role of nationality.

## Practical application

Subsequent research could deeply explore the original nationality background of athletes before naturalization, and analyze the performance differences of mixed-race athletes from dimensions such as genetic diversity, training resource allocation, sports immigration and cultural identity, to improve the existing research system. The findings of this study have important reference value for policymakers and coaches of endurance running projects in various countries: accurately understanding the influence of nationality, gender and age on running performance and their variation patterns with race distance helps to formulate development strategies that conform to national conditions and scientific training programs. In summary, this study provides an important basis for the theory and practice of endurance running projects through systematic analysis of multi-source data, and can provide theoretical reference for relevant policy formulation.

## Data Availability

The datasets generated during and/or analysed during the current study are available in the [OSF] repository, [https://osf.io/5fbma/overview?view_only=8181fb0b52fd41eeba155ecd39de7d62].
